# Supplementary material for: Time to non-adherence to iron and folic acid supplementation and associated factors among pregnant women in Hosanna town, South Ethiopia: Cox-proportional hazard model
Source: PLoS One. 2022 Sep 23;17(9):e0275086. doi: 10.1371/journal.pone.0275086 (PMC9506621; doi:10.1371/journal.pone.0275086)
Supplement: S1 Data — (DOCX) [file pone.0275086.s001.docx]

### **Questionnaire**

| Questionnaire identification number_______________  Interviewer name______________________ Date ____________signature__________________  Name of Kebele___________ | | | |
| --- | --- | --- | --- |
| Result:(For Office use 0nly)1.Completed 2.Partially completed 99.Others(specify)_____ | | | |
| Code | **Part one: Demographic and socioeconomic variables** | | Skip |
| 101 | How old are you? | _____________Years |  |
| 102 | Marital status | 1. Married  2. single  3. Divorced  4. widowed |  |
| 103 | Have you ever attended formal education? | Yes 2. No | 2→105 |
| 104 | What is the highest level of grade you completed: | ____________grade |  |
| 105 | What is your current Occupation? | 1. Government employ |  |
|  |  | 1. NGO employ |  |
|  |  | 1. Hired in private sector |  |
|  |  | 1. Merchant |  |
|  |  | 1. House wife |  |
|  |  | 1. Daily labourer |  |
|  |  | 99.Others(specify)________ |  |
| 106 | What is your religion? | 1. Protestant |  |
|  |  | 2. Orthodox |  |
|  |  | 3. Muslim |  |
|  |  | 4. Catholic |  |
|  |  | 99. Other(specify)_______ |  |
| 107 | Ethnicity | 1. Hadiya |  |
|  |  | 1. Kambata |  |
|  |  | 1. Silti |  |
|  |  | 1. Amhara |  |
|  |  | 99. other(Specify)_______ |  |
| 108 | HHs family Size | _____________ |  |
| Part Two: Does the household have any of the following properties? (check all appropriate box) | | | |
|  | No  Yes | | |
| 201 | Home ownership |  |  |
| 202 | Do you possess TV in your Residence? |  |  |
| 203 | Functioning CD player/ Radio/IPod/G-pass |  |  |
| 204 | An electric Mitad(an electrical machine to bake injera) |  |  |
| 205 | Electric stove / Gas Stove/Cylinder |  |  |
| 206 | Refrigerator(fridge) |  |  |
| 207 | Digital/video camera |  |  |
| 208 | washing machine |  |  |
| 209 | Bed with sponge/Spring mattress |  |  |
| 210 | Chair/Table |  |  |
| 211 | Chest drawer/ biffe/ comadienno |  |  |
| 212 | Sofa |  |  |
| 213 | Laptop/desktop computer |  |  |
| 214 | Non mobile phone |  |  |
| 215 | Mobile phone |  |  |
| 216 | Car |  |  |
| 217 | Taxi |  |  |
| 218 | Bajaj |  |  |
| 219 | Motor Cycle |  |  |
| 220 | Bicycle |  |  |
| 221 | An animal draw Cart |  |  |
| 222 | Bank or microfinance saving account |  |  |
| 223 | Cemented type of floor |  |  |
| Part Three: Obstetric Health related factors | | | |
| 301 | LNMP (If you don’t know, please Estimate your GA) | _______________ |  |
| 302 | Was the current pregnancy panned? | 1.Yes 2.No |  |
| 303 | Where do you make your ANC follow up (Possibly multiple options) | 1. Hospital 2. Health center 3. Health post 4. Private clinic |  |
| 304 | What was your gestational age at your first booking for ANC? | 1.________weeks  2._________months |  |
| 305 | Frequency of ANC visits | 1. only once 2. Twice times 3. three times 4. ≥4 times |  |
| 306 | Have you ever skipped your IFA ? | 1.Yes 2. No | 2→308 |
| 307 | If yes, why you skipped it? (Multiple options ) | 1. Forgetfulness 2. Travel 3. Constipation 4. Gastritis 5. Vomiting   99.Others(Specify)________ |  |
| 308 | How many pregnancy have you had so far? | ___________ | 1→314 |
| 309 | How many deliveries have you had so far? | ___________ |  |
| 310 | Have you had history of abortion? | 1.Yes 2. No |  |
| 311 | Have you had history of Still birth? | 1.Yes 2. No |  |
| 312 | Have you had history of preterm birth | 1.Yes 2. No |  |
| 313 | Previous low birth weight (LBW) | 1.Yes 2. No |  |
| 314 | Are you told to be anemic during current pregnancy? | 1.Yes 2. No |  |
|  | **Part Four: Personal exposure to media related factors** | |  |
| 401 | Frequency of on TV medical advice follow-up | 1. Not at all 2. Less than once 3. At least once a week |  |
| 402 | Frequency of listen to radio | 1. Not at all 2. Less than once 3. At least once a week |  |
| 403 | Frequency of reading medical magazine | 1. Not at all 2. Less than once 3. At least once a week |  |
|  | Part Five: Health facility related factors | |  |
| 501 | How long does it take to reach Health institution from your residence? | __________min |  |
| 502 | How long did it take to receive ANC service(min) in health institutions | __________min  2 ___________hr |  |
| 503 | Have you supplied with sufficient IFA when you visit health facility | 1.Yes 2. No |  |
| 504 | Did you get counseled on benefit of IFAS? | 1.Yes 2. No |  |
| 505 | Got counseled how often to take IFAS? | 1.Yes 2. No |  |
| 506 | Got counseled on how long to take IFAS? | 1.Yes 2. No |  |
| 507 | Got counseled on possible side effects | 1.Yes 2. No |  |
| 508 | Got counseled on managing side effects | 1.Yes 2. No |  |
| 509 | Got counseled on anemia | 1.Yes 2. No |  |
| Part Six: Knowledge on IFAS and Anemia | | | |
| 601 | Do you think IFAS during pregnancy helpful to the mother? | 1.Yes 2. No |  |
| 602 | IS IFAS during pregnancy helpful to the fetus? | 1.Yes 2. No |  |
| 603 | Do you think IFAS should begin upon confirmation of pregnancy? | 1.Yes 2.No |  |
| 604 | Do you think IFAS should continue throughout pregnancy? | 1.Yes 2. No |  |
| 605 | Do you think that IFAS prevent anaemia during pregnancy? | 1.Yes 2. No |  |
| 606 | Do you think IFAS should continue to postpartum period? | 1.Yes 2. No |  |
| 607 | Do know IFAS do not cause big baby delivery | 1.Yes 2. No |  |
| 608 | Do know that IFAS during pregnancy prevents birth defects? | 1.Yes 2. No |  |
| 609 | Does pregnancy make women anemic? | 1.Yes 2. No |  |
| 610 | Does anaemia cause shortness of breath? | 1.Yes 2. No |  |
| 611 | Does Anaemia cause weakness in pregnant women? | 1.Yes 2. No |  |
| 612 | Does anaemia manifested by pale skin or tongues? | 1.Yes 2. No |  |
| 613 | Do you know anaemia during pregnancy is preventable? | 1.Yes 2. No |  |
| 614 | Do you know preventive measures of anaemia | 1.Yes 2. No |  |
| 615 | Do you know the consequence anemia pregnancy? | 1.Yes 2.No |  |
| Part Seven: IFAS Timing and Adherence | | | |
| 701 | When did you begun to take your IFAS | _________________ |  |
| 702 | How many IFA pills have you taken in last seven days? | __________________ |  |
| 703 | How many IFAS pills have you taken till to day? | _________________ |  |
| 704 | To interviewer (Please verify it with pill count for consumed IFAS) | _______________ |  |

Thanks, this is the last!
